# Genetic variation for terminal heat stress tolerance in winter wheat

**DOI:** 10.3389/fpls.2023.1132108

**Published:** 2023-02-22

**Authors:** Jianming Fu, Robert L. Bowden, S. V. Krishna Jagadish, P. V. Vara Prasad

**Affiliations:** ^1^ Department of Agronomy, 2004 Throckmorton Plant Sciences Center, Kansas State University, Manhattan, KS, United States; ^2^ USDA–ARS Hard Winter Wheat Genetics Research Unit, 4008 Throckmorton Plant Sciences Center, Kansas State University, Manhattan, KS, United States; ^3^ Department of Plant & Soil Science, Texas Tech University, Lubbock, TX, United States

**Keywords:** heat stress, wheat, chlorophyll, yield components, controlled environment

## Abstract

In many regions worldwide wheat (*Triticum aestivum* L.) plants experience terminal high temperature stress during the grain filling stage, which is a leading cause for single seed weight decrease and consequently for grain yield reduction. An approach to mitigate high temperature damage is to develop tolerant cultivars using the conventional breeding approach which involves identifying tolerant lines and then incorporating the tolerant traits in commercial varieties. In this study, we evaluated the terminal heat stress tolerance of 304 diverse elite winter wheat lines from wheat breeding programs in the US, Australia, and Serbia in controlled environmental conditions. Chlorophyll content and yield traits were measured and calculated as the percentage of non-stress control. The results showed that there was significant genetic variation for chlorophyll retention and seed weight under heat stress conditions. The positive correlation between the percent of chlorophyll content and the percent of single seed weight was significant. Two possible mechanisms of heat tolerance during grain filling were proposed. One represented by wheat line OK05723W might be mainly through the current photosynthesis since the high percentage of single seed weight was accompanied with high percentages of chlorophyll content and high shoot dry weight, and the other represented by wheat Line TX04M410164 might be mainly through the relocation of reserves since the high percentage of single seed weight was accompanied with low percentages of chlorophyll content and low shoot dry weight under heat stress. The tolerant genotypes identified in this study should be useful for breeding programs after further validation.

## Introduction

Wheat (*Triticum aestivum* L.) is a major staple food crop and provides a significant portion of proteins and calories for the human population worldwide (FAO, 2012; Mishra et al., 2021). The world population growth requires wheat production increases. However, this demand is seriously challenged by high temperature stress in the wheat growing season, particularly in the grain filling stage, the terminal heat stress. Wheat originated in the Fertile Crescent area, a temperate region (Heun et al., 1997; Feldman and Levy, 2015), and is highly susceptible to high temperatures. Heat stress is often defined as the rise in temperature, usually 10–15 °C, beyond a threshold for a period of time sufficient to cause irreversible damage to plant growth and development (Wahid et al., 2007). Heat stress damages wheat plants in a number of different ways. At the molecular level, heat stress reduces the activities of enzymes, such as Rubisco activase and soluble starch synthase, which participate in the photosynthesis and starch biosynthesis (Keeling et al., 1993; Jenner, 1994; Mathur et al., 2014), changes membrane protein structural conformation, lipid composition, the timing and the abundance of gene expression that regulates grain development (Anderson and DuPont, 2003; Altenbach and Kothari, 2004; Wan et al., 2008), promotes the production of reactive oxygen species (ROS) and ethylene, and enhances the activities of enzymes, such as chlorophyllase, which participate in chlorophyll degradation (Todorov et al., 2003; Almeselmani et al., 2006; Hays et al., 2007). Heat stress reduces green (photosynthetic) area, accelerates senescence, shortens grain filling duration, increases photorespiration (Kuroyanagi and Paulsen, 1985; Cossani and Reynolds, 2012; Fu et al., 2012; Jagadish et al., 2016), causes male sterility (Anjum et al., 2008; Kaur and Behl, 2010), and increases the number of structurally abnormal and nonfunctional florets (Hedhly et al., 2009). Ultimately, these changes detrimentally affect yield components, such as reducing single grain weight or seed number. Wiegand and Cuellar (1981) reported that wheat grain filling duration shortened 3.1 days and single seed weight decreased 2.8 mg per degree Celsius increase in mean daily air temperature during grain filling period.

Based on the accumulated field yield data and weather data, detrimental effects of high temperature on wheat productivity were quantified at regional scales. A one degree Celsius temperature increase in mean air temperature was estimated to reduce wheat yield 3-17% in the Indo-Gangetic Plains in South Asia (Tiwari et al., 2013). Every 1°C increase in growing season temperature decreased wheat yield 3-10% in China (You et al., 2009). Every 1°C increase in average night temperature decreased wheat yield 10% in Mexico (Lobell et al., 2005). A 1°C increase in mean temperature was associated with a predicted decrease in wheat yield of 21% in Kansas USA (Barkley et al., 2013). Wheat yield could be reduced up to 50% when growing season temperature was two degrees Celsius higher than the average in Australia based on a simulation model study (Asseng et al., 2011). As climate change progresses and the Earth surface temperature gradually increases, heat episodes are also predicted to increase in frequency, length, severity and peak temperature (Intergovernmental Panel on Climate Change, 2015). Thus, the negative effects of high temperatures on the productivity of wheat and other crops will become more detrimental (Semenov and Halford, 2009; Lobell et al., 2011). Globally, wheat grain yield is estimated to be reduced by 6% for each degree Celsius of temperature increase (Asseng et al., 2015).

A sustainable way for mitigating heat stress damage is to develop tolerant varieties. Although several approaches, such as mutagenesis (Mullarkey and Jones, 2000), synthetics (Ratteya et al., 2011; Sareen et al., 2012) and transgenics (Fu et al., 2008), have been used in attempts to develop tolerant varieties, conventional breeding remains the most important approach which involves screening germplasm lines for identifying tolerant lines and then transferring the tolerance traits into commercial lines. Identifying lines with significant tolerance may require evaluating large numbers of germplasm lines in environment- and developmental stage-precisely controlled conditions. We have developed a protocol for such purposes, and recently we reported genetic variation of heat tolerance in a spring wheat diversity panel (254 lines) (Fu et al., 2022). The objectives of this work were to (1) investigate genetic variation and correlations of chlorophyll retention and yield components, and (2) identify genotypes with high retention of chlorophylls and/or seed weight in winter wheat under terminal heat stress conditions.

## Materials and methods

### Plant materials

The plant materials used in this study consisted of 304 winter wheat lines (**Supplemental Table 1**). Entries 1 to 301, 303 were Triticeae Coordinated Agricultural Project (TCAP) winter wheat lines from wheat breeding programs of public universities and private sectors across the United States of America. The origin information can be found at the T3 (The Triticeae Toolbox) site: https://wheat.triticeaetoolbox.org/. Entries 302 and 304 were from Serbia and Australia, respectively, and were previously reported to have capability of delayed chlorophyll degradation under terminal heat stress conditions (Alkhatib and Paulsen, 1990; Ristic et al., 2007).

### Plant growth conditions and heat stress treatment

Two experiments, Exp. 1 and Exp. 2, were conducted in the controlled environmental research facility at Kansas State University and USDA/ARS/Hard Winter Wheat Genetics Research Unit, Manhattan KS. In Exp. 1, seeds of 304 winter wheat lines were placed on wetted germination papers in plastic germination boxes and vernalized at 4°C for eight weeks. Six seedlings from each line were planted in pots containing Metro Mix 200 potting soil (Hummert International). One seedling was transplanted per pot. The pot diameter and the height was 7 and 25 cm, respectively. The plants were grown in the greenhouse. The greenhouse conditions were previously described (Fu et al., 2015). Briefly, plants were grown in a greenhouse at day/night temperature settings of 25/20°C with a photoperiod of 16 h. Natural light was supplemented with high pressure sodium lights to maintain the photoperiod and light levels. Humidity was not controlled nor monitored in the greenhouse. The control group was maintained under these conditions in the greenhouse for the duration of the experiment. Miracle Gro Fertilizer (Stern’s Miracle-Gro Products, Inc., Port Washington, NY) 24-8-16 (N-P-K) at a concentration of 0.5 g dry weight per liter was applied at the rate of 100 ml per pot once a week for the duration of the experiment, as previously described (Ristic et al., 2009). Insecticide Marathon 1% Granular (Imidacloprid, OHP, Inc., Bluffton, SC, USA) was applied once at the tillering stage to the soil mix according to manufacturer's instructions to control aphids and thrips. Elemental sulfur (99%) was applied in a sulfur vaporizer for two hours each night when powdery mildew was detected on plants. At anthesis each plant was tagged. Ten days after anthesis, three pots randomly selected from each genotype were moved to a Conviron Model E-36 growth chamber and exposed to heat stress for 16 d (36°C day, 30°C night, ± 1°C; RH 90 to 100%; photoperiod 16 h; PPFD of 480 μmol m^-2^ s^-1^). This greenhouse condition and the heating chamber condition were used based on the previous work that these conditions were previously successfully used for identifying heat tolerant wheat lines (Ristic et al., 2007; Fu et al., 2017; Fu et al., 2022). Pots were placed randomly in the growth chamber. To minimize dehydration of the leaf tissue during the heat treatment, pots were placed in trays containing standing water with an approximate depth of 1 cm. Following the heat stress treatment, plants were transferred back to the greenhouse until maturity for harvesting. The Conviron Model E-36 growth chamber had an internal floor dimension of 137 cm x 246 cm. Because this diversity wheat panel had a wide range of flowering time, one chamber had enough space for the heat treatment for 16 days for each plant.

Based on the data collected from Exp. 1, 10 heat tolerant lines and 3 susceptible lines (**Supplemental Table 2**) were selected for further validation before making crosses for developing mapping populations for genetic studies and before providing the tolerant lines to breeding programs. Our Exp. 1 was an individual pots- and heating chamber-based effort for evaluating more than 300 wheat lines for the terminal heat stress tolerance, and was very labor-intensive, particularly during the heat treatment time period. In Exp. 2, the 13 wheat lines selected based on the results from Exp. 1 were used (**Supplemental Table 2**). The experimental procedures and conditions including the heat treatment were the same as those in Exp. 1 except that ten plants of each line were grown in a growth chamber (25°C day, 20°C night, ± 1°C; RH 70%; photoperiod 16 h; PPFD of 480 μmol m^-2^ s^-1^) and half of them were used for heat treatment.

### Measurement of chlorophyll content

Chlorophyll content was measured using a self-calibrating SPAD chlorophyll meter (Model 502, Spectrum Technologies, Plainfield, IL) as described by Ristic et al. (2008) with modifications. Briefly, three readings were taken in the middle of the flag leaf of the primary tiller of each plant and averaged for every pot. The measurements were conducted at Days 0, 4, 8, 12, and 16 during the heat stress treatment.

### Harvesting and yield data

At maturity, plant parts above the soil surface were harvested. Spikes from all tillers were threshed, grains from all spikes were counted and weighed, and all tiller shoots were oven-dried at 65°C for 7 days and weighed, as previously described (Ristic et al., 2009).

### Statistical analysis

To compensate for innate differences in trait components, heat tolerance was quantified using the percentage of the control for each genotype. Pots were considered to be the experimental units. For each pot in the heat stress treatment, the chlorophyll retention at 4, 8, 12 or 16 days was expressed as a percentage of Day 0 (control). The rationale for using chlorophyll content at Day 0 as the control was previously described in Ristic et al. (2008), and this method was later on used by Sharma et al. (2017) and Fu et al. (2022). Briefly, the rationale stood on the observations that during the first 26 days after flowering the chlorophyll contents were not significantly changed under the control condition for hexaploid common wheat, tetraploid wheat and maize (Ristic et al., 2008). The rationale for using percentage of control rather than heat susceptibility index (HSI, Fischer and Maurer, 1978) was given in Fu et al. (2015), and this method was later on used by Fu et al. (2022); Fu et al. (2017). Briefly, the rationale stood on the facts including (1) that percent of control is always non-negative and a HSI can be negative in some cases and a negative number can cause problems in some statistical analyses such as PROC GLIMMIX which lacks convergence for a negative number, and (2) percent of control for each accession is more intuitive and is unaffected by the group of accessions under evaluation and, in contrast, HSI is the opposite (Fu et al., 2015). For the yield traits, percentage of control was calculated as follows: Percent of Control = 100 x (Y_h_/Y_c_).

where Y_h_ is the value for each experimental unit in the heat stress treatment and Y_c_ is the average value for the corresponding experimental units in the non-stress control condition. Higher mean percentage of control indicates greater heat tolerance. The experiment was analyzed as a completely randomized design. Data were obtained for two runs of the experiment (Exp. 1 and Exp. 2). Each experiment was analyzed separately. Genotype was analyzed as a fixed effect. Analysis of variance was performed with SAS 9.4 (SAS Institute, 2010) PROC GLIMMIX. The Gaussian (normal), Poisson, lognormal, and negative binomial distributions were tested for best fit on the basis of standard fit statistics and examination of the residuals. For each variable, the negative binomial distribution was used because it had the best fit. Least squares means for genotypes (lines) were back-transformed for presentation using the ilink function of GLIMMIX. Least squares means of genotypes were compared with an F test, and then separated using the Tukey-Kramer multiple comparison test. Correlations were calculated using SAS PROC CORR.

## Results

### Chlorophyll content

**Figure 1:**
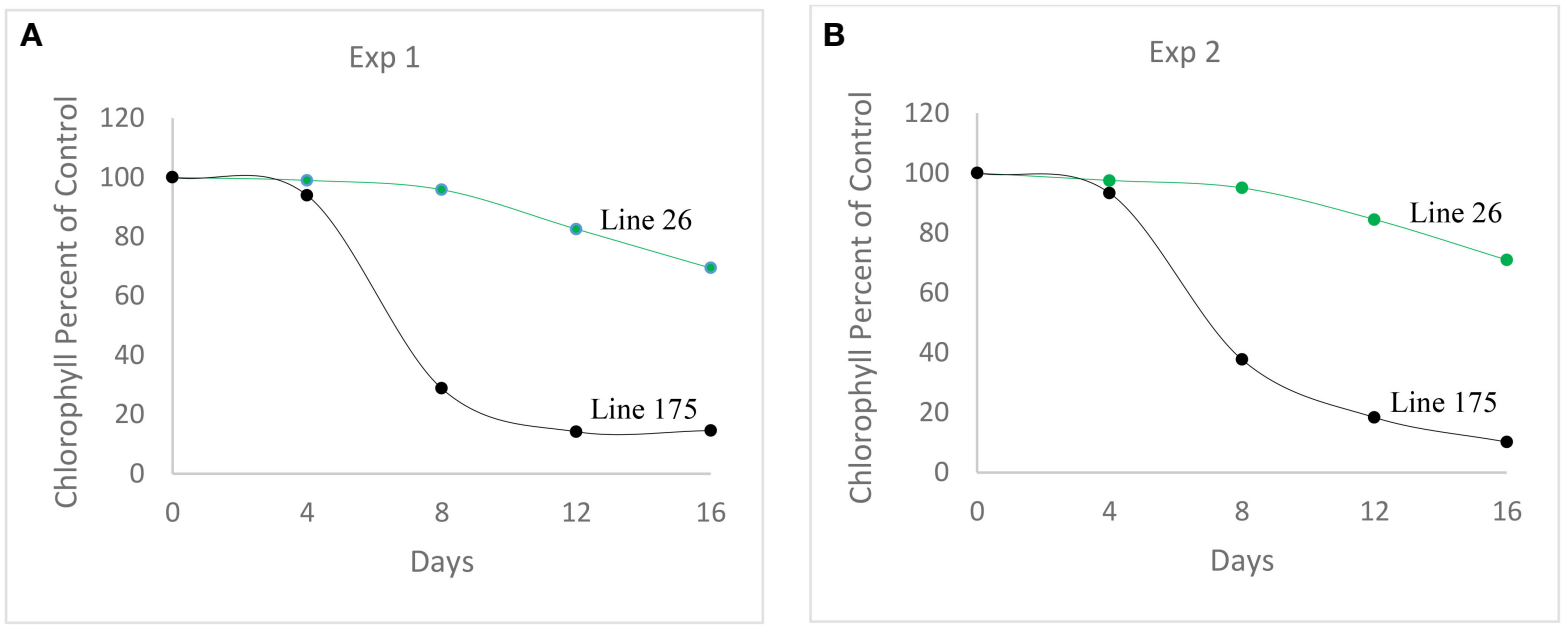
Chlorophyll retention rates in two contrasting genotypes. Tolerant Line 26 (OK05723W), with high chlorophyll retention and susceptible Line 175 (TX04M410164) with extremely low chlorophyll retention. **(A)** Exp. 1. **(B)** Exp. 2.

The percent of control for chlorophyll content at Day 4 of heat treatment among the winter wheat lines was significantly different in both Exp. 1 (F = 4.00, P < 0.0001) and Exp. 2 (F = 9.17, P < 0.0001). Similarly, the percent of control for chlorophyll content at Day 8, 12 and 16 of heat treatment among the winter wheat lines was significantly different in both Exp. 1 (F = 3.55 to 3.67, P < 0.0001) and Exp. 2 (F = 18.59 to 23.01, P < 0.0001) as well (**Table 1**). These results demonstrate that the genetic variation of the stay-green traits studied under the heat stress conditions among the winter wheat lines was significant. The percent of control for chlorophyll content at Day 16 (the last day) of heat treatment was used to determine the heat stress tolerance or susceptibility of each wheat line, and was subjected to multiple comparisons of the least square means using a conservative Tukey-Kramer method. The results were presented in **Supplemental Table 3** for Exp. 1 and the mean of all 304 lines was 26.4. The multiple comparison results for Exp. 2 were presented in **Supplemental Table 4**. The mean of all thirteen lines in Exp. 2 was 41.2. The mean of ten tolerant lines was 49.8, and the mean of three susceptible lines was 12.3. An example of one tolerant line (Line 26, OK05723W) and one susceptible line (Line 175, TX04M410164), in terms of percent for chlorophyll retention, was shown in **Figure 1**. Lines 26 and 175 showed 69.4% and 14.6% in Exp. 1 and 71% and 10.2% in Exp. 2 after 16 days of heat stress, respectively.

**Table 1:** Genotypic variation of percentage of control for stay-green and yield traits in winter wheat.

Traits	Exp.1		Exp. 2	
	F value†	P > F	F value†	P > F
Chlorophyll content Day 4	4.00	<.0001	9.17	<.0001
Chlorophyll content Day 8	3.55	<.0001	23.01	<.0001
Chlorophyll content Day 12	3.67	<.0001	18.59	<.0001
Chlorophyll content Day 16	3.66	<.0001	22.93	<.0001
Seed weight per plant	3.98	<.0001	4.06	0.0002
Single seed weight	4.77	<.0001	4.04	0.0002
Seed number	2.77	<.0001	1.12	0.3636
Shoot dry weight	2.55	<.0001	2.43	0.0143

Analysis of variance with SAS GLIMMIX using negative binomial distribution. Type III F test of fixed effects. In Exp. 1, DF equal 303; Denominator degrees of freedom equal 608 for chlorophylls and 607 for yield-related traits. In Exp. 2, DF equal 12; Denominator degrees of freedom equal 50.

### Seed weight per plant

The F values for the percent of control for seed weight per plant among the winter wheat lines were significant in both Exp. 1 (F = 3.98, P < 0.0001) and Exp. 2 (F = 4.06, P = 0.0002) (**Table 1**). Multiple comparisons for the percent of control for seed weight per plant were presented in **Supplemental Table 5** for Exp. 1. The mean across all 304 lines was 25% of the control. Multiple comparisons for the percent of control for seed weight per plant were presented in **Supplemental Table 6** for Exp. 2. The mean of all lines in Exp. 2 was 43.7% of the control. Line 175 was ranked second in both Exp. 1 (61.7% of control) and Exp. 2 (50.4%).

### Single seed weight

Similar to the seed weight per plant, the F values for the percent of control for single seed weight among the winter wheat lines were significant in both Exp. 1 (F = 4.77, P < 0.0001) and Exp. 2 (F = 4.04, P = 0.0002) (**Table 1**). Multiple comparisons for the percent of control for single seed weights were presented in **Supplemental Table 7** for Exp. 1. The mean across all 304 lines was 35.3% of the control. Line 175 (77.2% of the control) was ranked first in the top of the multiple comparisons. Multiple comparisons for the percent of control for single seed weights for Exp. 2 were presented in **Supplemental Table 8**. The mean of all thirteen lines was 52.3.3% of the control. The top two tolerant lines were Line 175 (64.4% of the control) and Line 15 (64.4%). Interestingly, Line 175 had low percent of control for chlorophyll content as illustrated earlier in **Figure 1**.

### Seed number

The analysis of variance revealed significant effects for genotypes for grain number per plant under heat stress expressed as a percentage of control in Exp. 1 (F = 2.77, P < 0.0001) (**Table 1**). Average grain number per plant under heat stress expressed as percentage across 304 lines in Exp. 1 was 68.8% (**Supplemental Table 9**). The F value for the percent of control for thirteen lines in Exp. 2 was not significant (F = 1.12, P = 0.3636) (**Table 1**).

### Shoot dry weight

Similar to the seed weight per plant and single grain weight, the Glimmix F values for the percent of control for shoot dry weights among the winter wheat lines were significant in both Exp. 1 (F = 2.55, P < 0.0001) and Exp. 2 (F = 2.43, P = 0.0143) (**Table 1**). The results of Tukey-Kramer multiple comparisons were shown and the means across all lines in Exp. 1 and Exp. 2 were 87.3% and 93.5% (**Supplemental Tables 10, 11**), respectively.

### Correlations

#### Correlation between traits

The correlations among the studied traits of chlorophyll content and yield components were presented in **Table 2**. Correlations of the percent of control for chlorophyll content among Days 4, 8, 12 and 16 were significant in both Exp. 1 (r = 0.2685 to 0.7215, P < 0.0001) and Exp. 2 (r = 0.4651 to 0.8477, P < 0.0001). Correlations for the percent of control between single seed weight and chlorophyll content at Day 4, 8 and 12 were significant in both Exp. 1 and Exp. 2. Correlations for the percent of control between shoot dry weight and seed weight per plant and seed number were significant in both Exp. 1 and Exp. 2. As expected, correlations among single seed weight, seed number and seed weight per plant were significant in both Exp. 1 and Exp. 2.

**Table 2:** Correlations among percentage of control for chlorophyll content, seed weight per plant, single kernel weight, seed number, and total shoot weight for Exp. 1 (above diagonal) and Exp. 2 (below diagonal).

Traits	Chlorophyll Day 4	Chlorophyll Day 8	Chlorophyll Day 12	Chlorophyll Day 16	Seed Weight per Plant	Single Seed Weight	Seed number	Shoot Dry Weight
**Chlorophyll Day 4**	1.0000	0.7215 <.0001	0.4663 <.0001	0.2685 <.0001	0.1592 <.0001	0.1852 <.0001	0.0939 0.0045	0.0957 0.0038
**Chlorophyll Day 8**	0.7113 <.0001	1.0000	0.6721 <.0001	0.3973 <.0001	0.1861 <.0001	0.2161 <.0001	0.1047 0.0015	0.0784 0.0180
**Chlorophyll Day 12**	0.5342 <.0001	0.8349 <.0001	1.0000	0.6173 <.0001	0.0561 0.0906	0.1271 0.0001	-0.0191 0.5584	0.0035 0.9150
**Chlorophyll Day 16**	0.4651 0.0001	0.7426 <.0001	0.8477 <.0001	1.0000	0.0335 0.3121	0.0977 0.0032	-0.0260 0.4327	-0.0280 0.3983
**Seed Weight per plant**	0.1396 0.2753	0.1593 0.2125	0.1568 0.2198	0.1050 0.4127	1.0000	0.7527 <.0001	0.7775 <.0001	0.2328 <.0001
**Single Seed Weight**	0.3537 0.0045	0.2544 0.0442	0.2485 0.0496	0.0826 0.5199	0.3087 0.0138	1.0000	-0.2234 <.0001	0.0193 0.5076
**Seed number**	-0.1046 0.4144	-0.0168 0.8959	0.0028 0.9825	0.0533 0.6784	0.7768 <.0001	-0.3395 0.0065	1.0000	0.3503 <.0001
**Shoot Dry Weight**	-0.2263 0.0746	-0.1510 0.2376	-0.0984 0.4429	0.0246 0.8480	0.4680 <.0001	-0.4790 <.0001	0.7903 <.0001	1.0000

For each cell, upper number is the Pearson correlation coefficient (r), and lower number is the P value for the null hypothesis r = 0. In Exp.1 n=912. In Exp.2 n=63.

#### Correlation between two experiments

**Figure 2:**
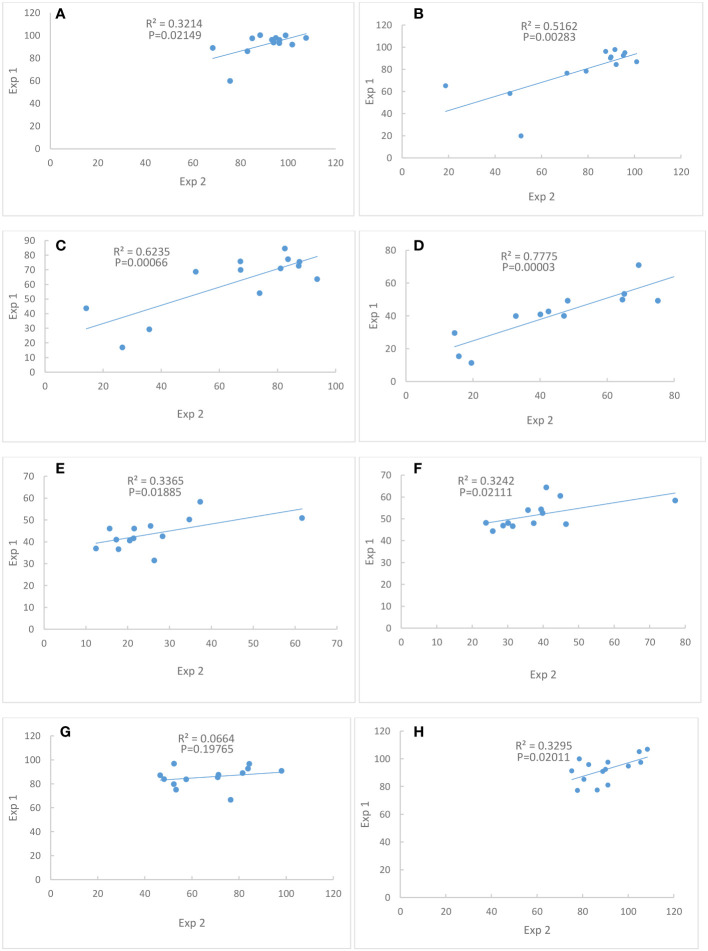
Correlations of percent of control between Exp. 1 and Exp. 2. **(A)** Chlorophyll content at Day 4. **(B)** Chlorophyll content at Day 8. **(C)** Chlorophyll content at Day 12. **(D)** Chlorophyll content at Day 16. **(E)** Seed weight per plant. **(F)** Single seed weight. **(G)** Seed number. **(H)** Shoot dry weight. N = 13.

Correlations between Exp. 1 and Exp. 2 were significant for traits chlorophyll content at Days 4, 8, 12 and 16, seed weight per plant, single seed weight and shoot dry weight (**Figure 2**), indicating that the performances of these traits were consistent between the two experiments. However, the correlation of seed number between Exp. 1 and Exp. 2 was not significant (**Figure 2**).

## Discussion

Heat stress, particularly terminal heat stress, damages many different aspects of wheat plants, and consequently reduces the grain yield. Using conventional breeding to develop tolerant varieties remains the best way to mitigate heat damage. Identifying genotypes (lines) with tolerance traits is required for breeding programs. Growth chamber-based and field-based efforts for identifying tolerant genotypes were previously reported (Asana and Williams, 1965; Wardlaw, 1994; Dias and Lidon, 2009; Banyai et al., 2014; Bergkamp et al., 2018; Rezaei et al., 2018; Sebela et al., 2020). However, in the chamber-based studies on the terminal stress only a relatively small number of genotypes were typically evaluated. One drawback of such efforts is that the tolerant genotypes selected may have limited tolerance due to a small pool of genotypes available for the selection. In the field-based studies although a large number of genotypes can be planted there are a number of problems. First, a large panel of diverse genotypes is intrinsically associated with a large variation of phenological traits, e.g., flowering time, and it is almost impossible to compare chlorophyll contents at the same development stage under the same environmental conditions among genotypes. Second, the instability of heat stress imposition under field conditions would lead to errors in phenotypic data and later translated the errors into genetic analysis. Third, it may be difficult or impossible to separate effects of heat stress from other stresses, such as drought when sufficient irrigation capability is lacking. Thus, there are advantages to testing a large genetically diverse germplasm collection under precisely controlled conditions. Our protocol has the capability to evaluate more than 300 wheat lines for terminal heat stress tolerance in chamber-based conditions, thus overcoming the problems under field conditions.

In this study we evaluated the terminal heat stress tolerance of a diversity panel consisting of 304 winter wheat lines. The results showed that genetic variation for chlorophyll retention and grain weights were significant, and the correlation between the percent of chlorophyll content and the percent of single seed weight was significant as well. Some genotypes, such as Line 26, with high percent of single seed weight, also had high percent of chlorophyll retention and high percent of shoot dry weight. This might be explained by grain filling being mainly contributed from the current photosynthetic products. However, some genotypes, such as Line 175, with high percent of single seed weight, had low percent of chlorophyll retention during the grain filling stage and low percent of shoot dry weight. This might be explained by grain filling being mainly contributed from the relocation of soluble sugars in the stem reserve. These two proposed scenarios represent two different grain filling mechanisms, and after further confirmation and validation introgression of different grain filling mechanisms into the same variety may substantially reduce wheat grain yield loss under heat stress conditions.

Our findings are in agreement with several previous studies which reported that stay-green was significantly correlated with the grain yield under heat stress environmental conditions in wheat (Hede et al., 1999; Kumar et al., 2010; Lopes and Reynolds, 2012; Nawaz et al., 2013; Kumari et al., 2013). The significant correlation between the stay-green and the grain yield under heat stress conditions is the rationale for selecting functional stay-green genotypes to develop heat-tolerant varieties. The importance of stem reserve remobilization for grain filling for some wheat genotypes under heat stress conditions was reported previously (Blum et al., 1994; Blum, 1998), and thus introgression of this trait may improve wheat grain yield under heat stress conditions.

In this work, although the percent retention of chlorophyll and the percent of single seed weight were significantly correlated, the correlation was low. One possible cause for the low correlation was that heat stress damages sink (grain filling) components. For example, heat stress decreases starch synthase activity, and consequently limits starch synthesis and grain growth in wheat (Keeling et al., 1993). Another cause might be that for some genotypes grain filling was mainly contributed from the remobilization of stem reserves under heat stress conditions (Blum et al., 1994; Blum, 1998; Gebbing and Schnyder, 1999). The third cause might be that for some genotypes only small portion of current photosynthetic products was transported (partitioned) to the developing grains under heat stress conditions. The fourth cause might be that for some genotypes stay-green might be non-functional or partly functional. In this study, we did not measure the starch synthase activity, nor the photosynthesis of green tissues in heat stress, nor the actual remobilization of stem reserve in heat stress, and so these questions remain to be answered, and deserve separate investigations in the future. Interestingly, it appears that the correlations between the percent of control for single seed weight and the percent of chlorophyll retention were greater at the early stages of the heat stress than at the later stages of the heat stress. This may indicate that the current photosynthates might be transported to the developing grains more efficiently at the early stages of the heat stress than at the later stages of the heat stress. Although the correlation between the seed weight/plant and the single seed weight was lower (r = 0.3087, P < 0.0138) than the correlation between the seed weight/plant and the seed number (r = 0.7768, P < 0.0001) in Exp. 2, they were very similar (r = 0.7775, P < 0.0001; r = 0.7527, P < 0.0001) in Exp. 1. It is unclear whether the different panel composition or different experimental conditions during grain filling might account for the difference.

In conclusion, significant genetic variation for chlorophyll retention and grain yield-related traits exists under heat stress conditions in winter wheat. Heat-tolerant genotypes were identified and two possible grain filling mechanisms were proposed. Tolerant lines identified in this study were provided to breeding programs for developing heat-tolerant varieties and used for developing mapping populations for QTL mapping and fine mapping to locate genes responsible for heat tolerance.

## Data availability statement

The original contributions presented in the study are included in the article/**Supplementary Material**. Further inquiries can be directed to the corresponding author.

## Author contributions

JF, RB and PP designed experiments. JF conducted experiments and collected data. JF and RB did data analysis. JF prepared the manuscript. RB, SJ and PP reviewed and edited the manuscript. All authors contributed to the article and approved the submitted version.
